# Balloon-assisted Tracking of Guide Extension Catheter: A Novel Technique to Retrieve a Carotid Embolic Protection Device

**DOI:** 10.7759/cureus.4045

**Published:** 2019-02-11

**Authors:** Srilekha Sridhara, Siamac Yazdchi, Jamal T Alkhatib, Anna Berry, Ismail A. H Bokhari

**Affiliations:** 1 Internal Medicine, Banner Heart Hospital, Mesa, USA; 2 Internal Medicine, Banner University Medical Center, Phoenix, USA; 3 Cardiology, Kingman Regional Medical Center, Kingman, USA

**Keywords:** embolic prevention device, device retrieval, carotid artery stenting

## Abstract

Embolic protection devices are used to minimize the risk of stroke by preventing the migration of emboli during carotid artery stenting (CAS). After the successful conclusion of the CAS procedure, these devices are meant to be retrieved. Sometimes retrieval of the filter can be difficult. This difficulty in retrieval can be due to multiple factors such as incomplete stent expansion, stent fracture, vasospasm, and vessel tortuosity causing pseudostenoses.

In this case report, an under-expanded proximal carotid stent strut contributed to the filter not being retrievable in spite of maneuvers to retrieve the filter. An innovative approach was used; a coronary guide extension catheter was used to enhance support and balloon-assisted tracking of the extension catheter then permitted advancement of the retrieval device and ultimate retrieval of the filter. This technique to retrieve a carotid filter has not been previously described in the literature.

## Introduction

Carotid artery stenting (CAS) is the preferred treatment for patients with symptomatic carotid stenosis of 70% to 99% when clinical co-morbidities or anatomical factors make the patient at high risk for carotid endarterectomy (CEA). The 30-day risk for stroke is higher with stenting than with CEA. CAS is associated with a lower risk of myocardial infarction, cranial nerve palsy, and access-site hematoma compared to CEA. One of the ways to minimize the risk for stroke with CAS is the use of distal protection devices. The most commonly used device is the Emboshield filter (Abbott Vascular, Santa Clara, CA).

This case highlights how cross-specialty collaboration can help solve clinical conundrums and technical challenges. The challenge encountered in this case was difficulty in retrieving embolic filter device which got lodged in the left internal carotid artery. Knowledge of transradial angiography for coronary intervention where balloon-assisted tracking is frequently used for advancing guide catheters through vascular tortuosity was transferred and used in this carotid intervention. Balloon-assisted tracking over a guide extension catheter was performed to bail the operator out and retrieve the embolic filter device.

## Case presentation

A 71-year-old male with a history of hypertension, hyperlipidemia, chronic obstructive pulmonary disease, pre-diabetes, chronic smoking, and alcoholism presented with amaurosis fugax. He had suffered a previous left occipital lobe infarct. Carotid duplex and computerized tomography angiography (CTA) of the head and neck confirmed a high-grade left internal carotid artery stenosis (ICA). A five French (Fr) sheath was used for femoral access and aortic arch angiography was performed. Selective cannulation of the left common carotid artery (CCA) was done with an AR-1 catheter. Diagnostic angiography confirmed the noninvasive findings of a high-grade lesion in the left ICA (Video 1).

**Video 1:** Diagnostic angiography showing high grade left ICA lesion ICA: internal carotid artery

A seven Fr Cook Shuttle (Cook) sheath was advanced into left CCA over a glide wire advantage (Terumo). The Accunet embolic protection device (EPD) was deployed and pre-dilatation of the lesion was performed with a 4-mm balloon Maverick (Video 2).

**Video 2:** Pre-dilatation of ICA lesion

An Acculink 7 x 10 x 40-mm stent was deployed without difficulty (Video 3).

**Video 3:** Deployment of Acculink stent

Post-dilatation was performed with a 5-mm balloon (Video 4).

**Video 4:** Post-dilatation of the lesion/stent with 5mm balloon

Attempts to advance the EPD retrieval system beyond the origin of left ICA were unsuccessful. Even a smaller balloon was unsuccessful in crossing the under expanded proximal stent strut. Maneuvers like having the patient turn his head to the right were unsuccessful in advancing the EPD retrieval catheter.

After multiple such futile attempts, a concept that is routinely used in transradial percutaneous coronary intervention (PCI) came handy. The shuttle sheath was extended with a guide extension catheter to enhance support. A 2.5-mm balloon was advanced into this guide extension catheter (Video 5).

**Video 5:** 2.5mm balloon advanced into guide extension catheter

Balloon-assisted tracking of the guide extension catheter was performed into the left ICA beyond the point where the retrieval catheter was hanging up (Video 6).

**Video 6:** Balloon assisted tracking of guide extension catheter into left ICA

After this, advancing the retrieval catheter into the guide extension was easy and the filter was removed uneventfully.

Due to some focal weakness in the left upper extremity, a CTA of head and neck was performed which showed a small hemorrhagic transformation within the previous right occipital infarct. On discharge, the patient recovered completely and left the hospital with no neurological deficits.

## Discussion

The 30-day risk for stroke is higher with carotid stenting than with CEA [1]. To reduce stroke risk, there is a need for an embolic protection device during CAS. Theron J et al. performed the first carotid artery angioplasty with a distal balloon occlusion system EPD in 1990 [2]. Studies have demonstrated EPD use leads to a decrease in 30-day stroke rate [3]. The three categories of EPDs available are flow preservation devices, distal occlusion devices, and proximal protection devices. Table 1 reviews the two types of distal protection devices and their pros and cons.

**Table 1:** Distal protection devices

Filters:	Balloons:
1. Preserve antegrade flow	1. Antegrade flow interrupted
2. Used in the majority of cases worldwide	2. Preferred in pre-occlusive lesions
3. May get clogged	3. Do not permit angiography due to stagnant contrast column
4. Permit passage of small-sized microparticles depending on the pore size of the filter	4. Risk of intolerance when collateral circulation is inadequate
5. Can cause carotid dissection, embolize, and break or tear	5. Can cause carotid dissection, and could tear, embolize or break

Proximal protection devices are recommended for thrombus-containing lesions or in severely symptomatic lesions where even minimal manipulation of the lesion while initial wiring can cause a substantial risk for stroke. However, their downside is the risk of CCA injury, cerebral blood flow reversal, carotid vacuum effect leading to elongation, and constriction of deployed stent and their large profile [1]. 

Based on a large retrospective multi-center study, the use of EPDs is associated with a lower risk of adverse procedural events and there was no significant difference in adverse procedural risks between the different devices and types [4]. The device used in this case was the Accunet distal filter (Abbot Vascular, Santa Clara, CA). Distal filters offer only selective protection as particles <100 um can escape [1]. All EPDs are associated with vasospasm, arterial dissection, and equipment failure and run the risk of difficult retrieval. 

Techniques to retrieve trapped filters have included external carotid compression, rotation of the head to the contralateral side to change carotid angulation, and use of different catheters like the vertebral and JR-four [5-8]. In this case, none of these techniques were successful. Thinking outside the box enabled the operators to use a technique common in transradial angiography and apply it effectively. This saved the patient the need for open surgery to retrieve the filter, which has also been described as a last resort [9-10]. If the filter were to break off and migrate to the intracranial circulation, conservative management may be the only option [11].

Appropriate patient selection and choosing an appropriate EPD for an individual case can mitigate the risk of retained devices. Calcified plaques and tortuosity index >80° are associated with difficult retraction. Three-dimensional imaging can help in identifying potentially tricky situations. Difficult retrievals of EPDs lead to significantly longer procedural times and higher complications [12].

## Conclusions

EPDs are mandatorily required to be used with CAS to decrease stroke during CAS but carry an inherent risk of micro emboli mediated stroke, transient ischemic events, cognitive decline, vascular injury, difficult retrieval. Thorough knowledge of rescue and salvage techniques with EPD complications is critical. Cross-pollinated knowledge between different specialties can help disseminate these tricks and techniques. 
